# Treatment of Whooping Cough by Enemeta of Assafœtida

**Published:** 1857-08

**Authors:** 


*Treatment of Whooping Cough by Enemeta of Asafœtida.*—M. Ancelon recommends as the most reliable and efficient remedy in this distressing and obstinate affection, Enemeta of Assafœtida. "He declares it to be a reliable and an heroic remedy, in the second and third periods of the disease. Little can be expected from it in the first period. Much of its efficacy will depend on the dose and mode of administration. To Infants eighteen months to two years old, three injections should be directed, each of them containing in the smallest possible quantity of vehicle fifteen grains of asafœtida, and two drops of *Sydenham's Liqnid Laudanum* (*North American Medico Chirurgical Review.*)

